# Advancements and outcomes of robotic-assisted surgery in pediatric patients: a multicenter analysis

**DOI:** 10.3389/fped.2025.1652840

**Published:** 2025-09-08

**Authors:** Girolamo Mattioli, Giulia Rotondi, Stefano Avanzini, Michele Torre, Gaia Brenco, Carmelo Romeo, Francesco Molinaro, Marco Prestipino, Maurizio Cheli, Francesco Lacanna, Gabriella Paoli, Silvano Cincotti, Chiara Tombetti, Barbara Razore, Laura Oddera, Maria Beatrice Damasio, Andrea Michele Gino Maria Wolfler, Giuseppe Spiga, Federico Palo

**Affiliations:** ^1^ Department of Pediatric Surgery, IRCCS Istituto Giannina Gaslini, Genoa, Italy; ^2^ DINOGMI, University of Genoa, Genoa, Italy; ^3^ Unit of Pediatric Surgery, Department of Human Pathology of Adult and Childhood “Gaetano Barresi”, University of Messina, Messina, Italy; ^4^ Pediatric Surgery, Department of Medical, Surgical and Neurological Sciences, S. Maria Alle Scotte Hospital, University of Siena, Siena, Italy; ^5^ Division of Pediatric Surgery, General Hospital of Perugia, Perugia, Italy; ^6^ Department of Pediatric Surgery, ASST Papa Giovanni XXIII Hospital, Bergamo, Italy; ^7^ Ligurian Regional Health Service (ALISA), Genoa, Italy; ^8^ DIME-DOGE, Università di Genova, Genova, Italy; ^9^ Department of Clinical Engineering, IRCCS Istituto Giannina Gaslini, Genoa, Italy; ^10^ Department of Radiology, IRCCS Istituto Giannina Gaslini, Genoa, Italy; ^11^ Department of Emergency, Division of Anesthesia, IRCCS Istituto Giannina Gaslini, Genoa, Italy; ^12^ Department of Planning, Control and Quality Management, IRCCS Istituto Giannina Gaslini, Genoa, Italy

**Keywords:** robotic pediatric surgery, pediatric surgery, minimally invasive surgeries (MIS), surgical outcomes, technological innovation

## Abstract

**Background:**

Robotic surgery (RS) has gained widespread adoption in adult surgical specialties but faces unique challenges in pediatric applications due to anatomical, technical, and economic factors. This multicenter study evaluates the feasibility, clinical outcomes, and organizational implications of implementing robotic-assisted surgery in pediatric patients across various surgical disciplines.

**Methods:**

Data were retrospectively collected from 569 pediatric patients undergoing RS between 2015 and 2016 and 2020–2025 at five Italian pediatric surgery centers. Procedures included urological, gastrointestinal, oncological, and thoracic surgeries. Patient demographics, operative details, complications, and conversion rates were analyzed. Standardized operating room setups and trocar placements were developed to optimize surgical ergonomics.

**Results:**

The median patient age was 73.8 months (range 4–575.3 months), with 9.3% weighing less than 10 kg. Urological procedures comprised 47.2% of cases, with a 0.7% conversion rate to open surgery. Gastrointestinal, oncological, and thoracic procedures had conversion rates of 3.9%, 27.2%, and 13.5%, respectively. Postoperative major complications occurred in 7% of cases. The study confirmed the safety and feasibility of RS even in patients with significant clinical complexity (20.5% ASA ≥3). Robotic technology provided enhanced precision, dexterity, and visualization, enabling complex reconstructions in confined anatomical spaces. Centralized care in dedicated pediatric centers was critical for successful implementation.

**Conclusions:**

Robotic surgery represents a significant advancement in pediatric minimally invasive surgery, offering clinical benefits across multiple specialties. Despite economic and logistical challenges, centralization and dedicated multidisciplinary teams are essential to optimize outcomes and ensure safe, sustainable adoption of robotic techniques in pediatric surgery.

## Introduction

Robotic surgery (RS) has become increasingly widespread, particularly among adult urologists and gynecologists (1–3). However, since its introduction in 2001, its adoption in pediatric practice has faced significant challenges (4). These challenges encompass the complex development of pediatric-specific instruments, the need for a fundamental shift in the surgical team's approach, and the substantial economic and ethical considerations that must be addressed.

RS represents the advancement of minimally invasive surgery (MIS), offering all the benefits associated with this approach, including reduced operative trauma, lower postoperative pain, decreased need for analgesic medications, shorter hospital stays, faster recovery, and improved aesthetic outcomes (5, 6).

Numerous published studies and reports demonstrate the feasibility of RS, particularly in pediatric urology. Over time, the use of robotic systems in pediatric surgery has expanded to include a variety of procedures, extending beyond urology to encompass gastrointestinal (GI), thoracic, and hepatic surgeries (7–11).

RS overcomes some limitations of laparoscopic and thoracoscopic procedures by using da Vinci™ EndoWrist® technology (Intuitive Surgical Inc., Sunnyvale, California), which allows 7 degrees of freedom compared to the 4 degrees of laparoscopic instruments. RS also provides enhanced precision with a movement scaling of up to 5:1, eliminating the surgeon's postural tremor at the console (6, 11). This is complemented by high-resolution imaging from the endoscope, offering three-dimensional visualization and magnification up to 10–15 times. Moreover, the incorporation of fluorescence technology (Firefly®, Novadaq Technologies, Mississauga, ON) significantly improves the visualization of vascular structures, aiding in more precise surgical dissection (12, 13).

With enhanced precision and dexterity, robotic surgery proves ideal for complex procedures in confined spaces. Despite its established benefits in both adult and pediatric settings, economic challenges have limited its broader implementation in pediatric care (8, 10, 14).

This study investigates the feasibility and organizational implications of implementing robotic surgery in pediatric hospitals. Particular attention is given to the assessment of clinical advantages, infrastructural requirements, staff training needs and the integration of robotic systems within existing workflows. The objective is to offer a comprehensive analysis to support strategic decision-making for the safe, efficient, and sustainable adoption of robotic-assisted surgical technologies in pediatric care settings.

## Materials and methods

### Study period, inclusion/exclusion criteria, and surgical procedures

Patients who underwent robotic surgery between February 2015 and April 2016 and from March 2020 and May 2025, were included in the study.

This multicenter study was coordinated by the Department of Pediatric Surgery at IRCCS Giannina Gaslini in Genoa. The clinical data were collected from four additional Pediatric Surgery centers across Italy: Papa Giovanni XXIII Hospital in Bergamo, Santa Maria Della Misericordia Hospital in Perugia, Santa Maria alle Scotte University Hospital in Siena, and G. Martino University Hospital in Messina.

Newborns under 28 days of age were excluded from robotic surgery eligibility.

Surgical indications included both standard minimally invasive procedures and those more technically demanding, as determined by the surgeon's expertise and based on disease-specific assessments and multidisciplinary discussions. Most of the procedures were performed by a single surgeon, with assistance from junior consultants to assess the learning curve. The procedures were categorized into gastrointestinal (GI) surgery, urology, oncology, and thoracic surgery.

Data from some of the collaborating centers were partially incomplete; the statistical analysis was conducted taking this limitation into account.

### Informed consent and ethics committee approval

The Regional Ethics Committee approved the study protocol. The study protocol for each category of procedures was approved by the corresponding institutional ethics committee. Details of the project, surgical indications, potential risks and benefits, and technical aspects of RS were first discussed within a multidisciplinary board and subsequently with the patient and/or their legal guardians. Informed consent was obtained for all surgical procedures.

### Operating room (OR) and patient setting

**Figure 1:**
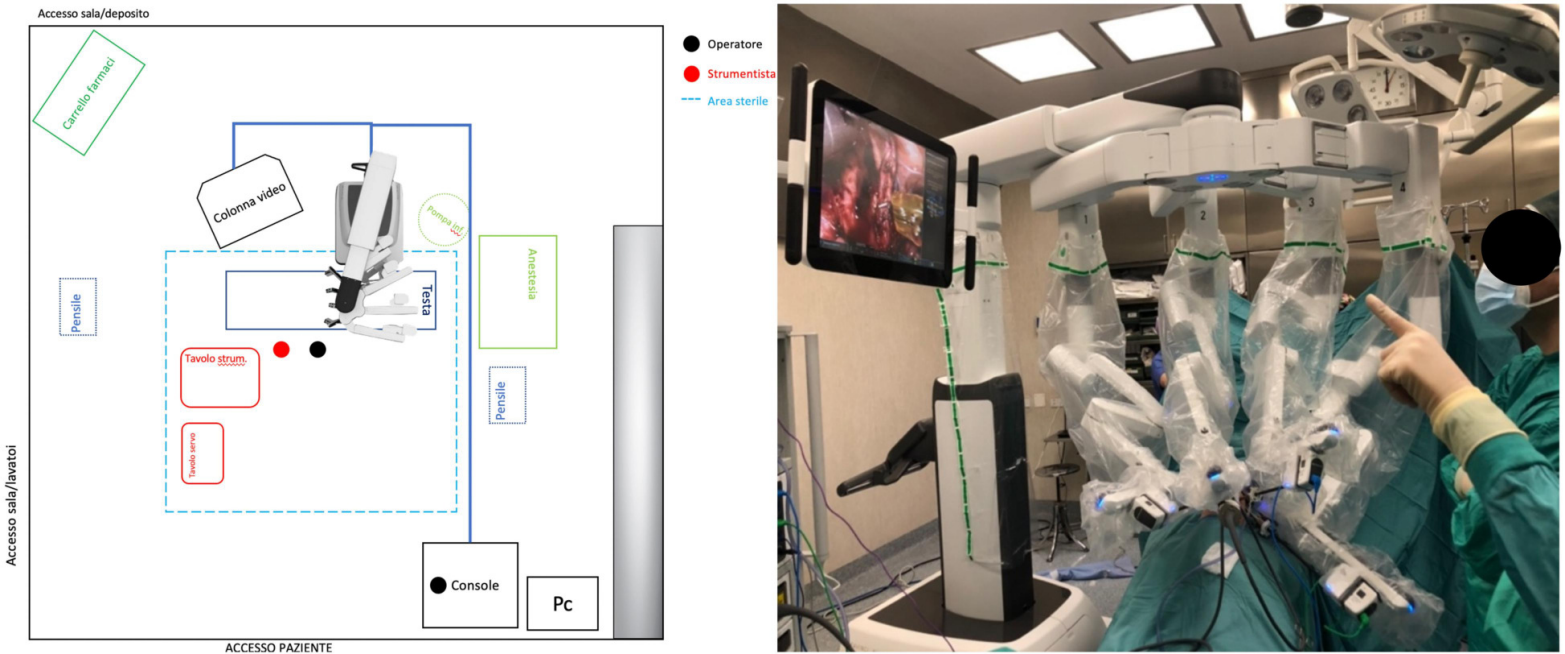
On the left, the planimetry (scale 1:36 cm) of the operating room for chest and airway procedures is shown (black dots indicate the surgeons; the red dot indicates the scrub nurse). The robot is positioned at the center of the bed and to the right of the patient, with the boom rotated 90 degrees clockwise. The video monitor is placed at the lower end of the bed, also to the right of the patient. On the right, an intraoperative photograph illustrates the actual layout of the room, as previously planned.

**Figure 2:**
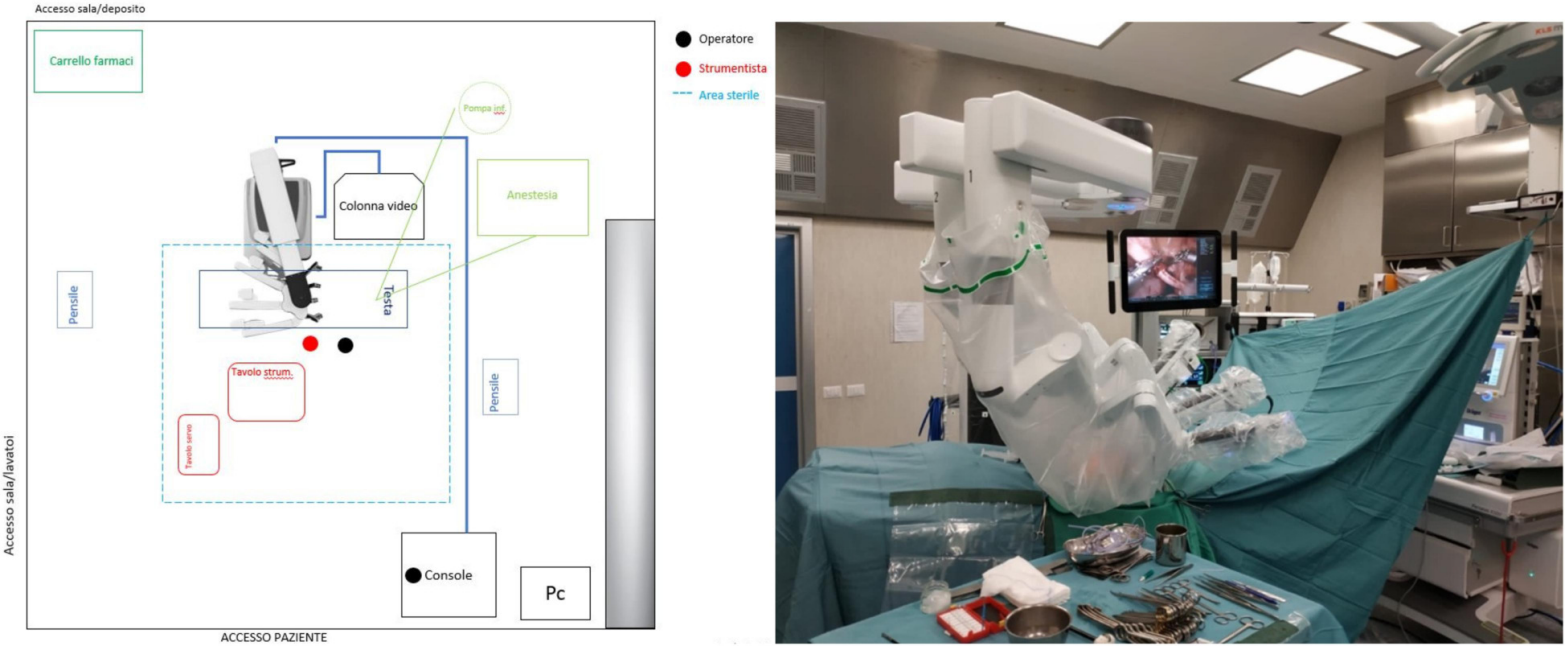
On the left, the planimetry (scale 1:36 cm) of the operating room for lower abdominal procedures is shown (black dots indicate the surgeons; the red dot indicates the scrub nurse). The robot is positioned at the center of the bed and to the right of the patient, with the boom rotated 90 degrees counterclockwise. The video monitor is placed at the upper end of the bed, also to the right of the patient. On the right, an intraoperative photograph illustrates the actual room layout as previously planned.

**Figure 3:**
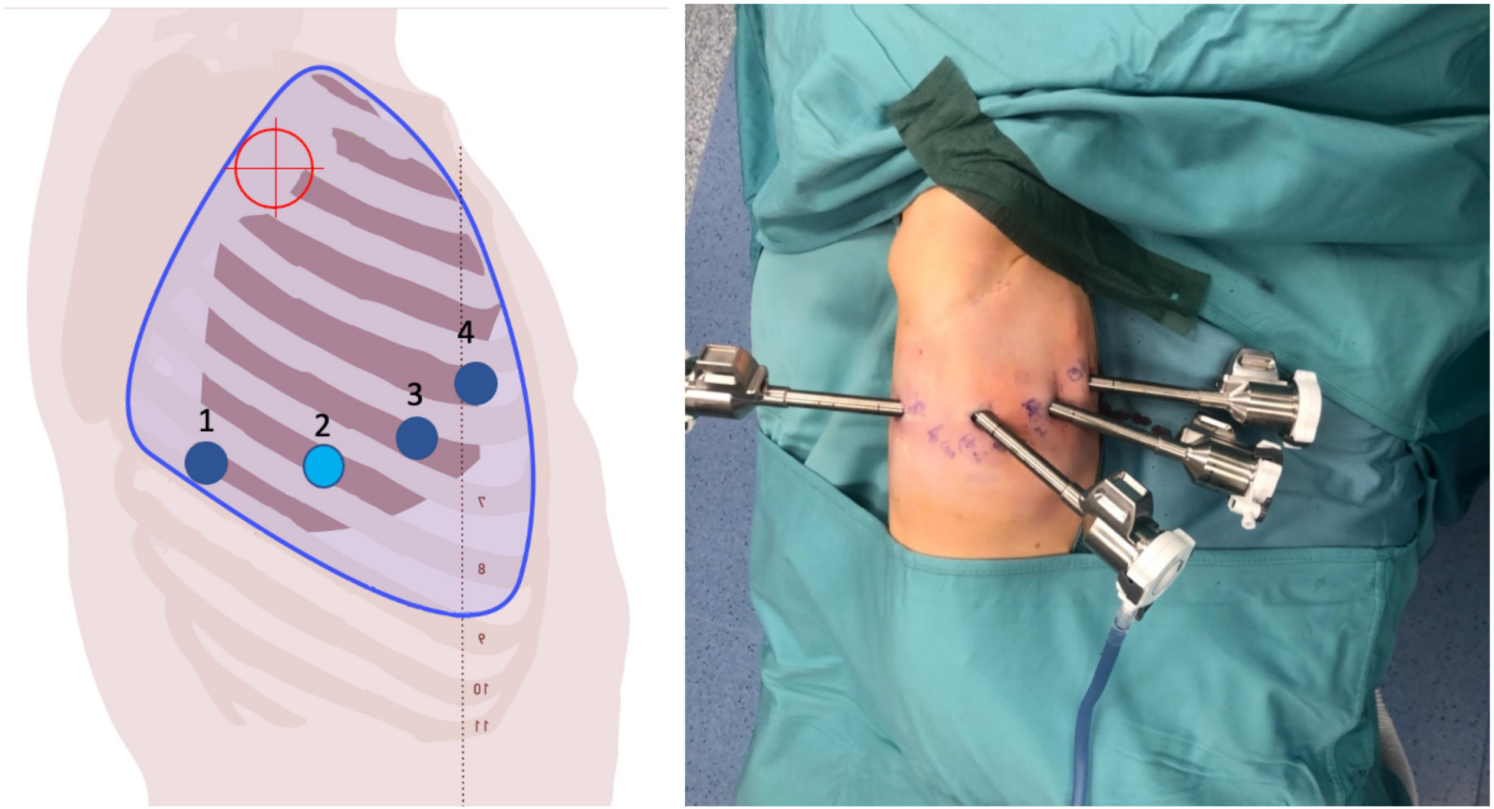
On the left, the port placement for tracheal or intrathoracic esophageal surgery is shown. In this specific case, the trocars are positioned across three different intercostal spaces (the light blue dot indicates the camera, the dark blue dots represent the operating instruments, and the red target marks the surgical objective). On the right, intraoperative photographs show the trocars in place prior to docking.

**Figure 4:**
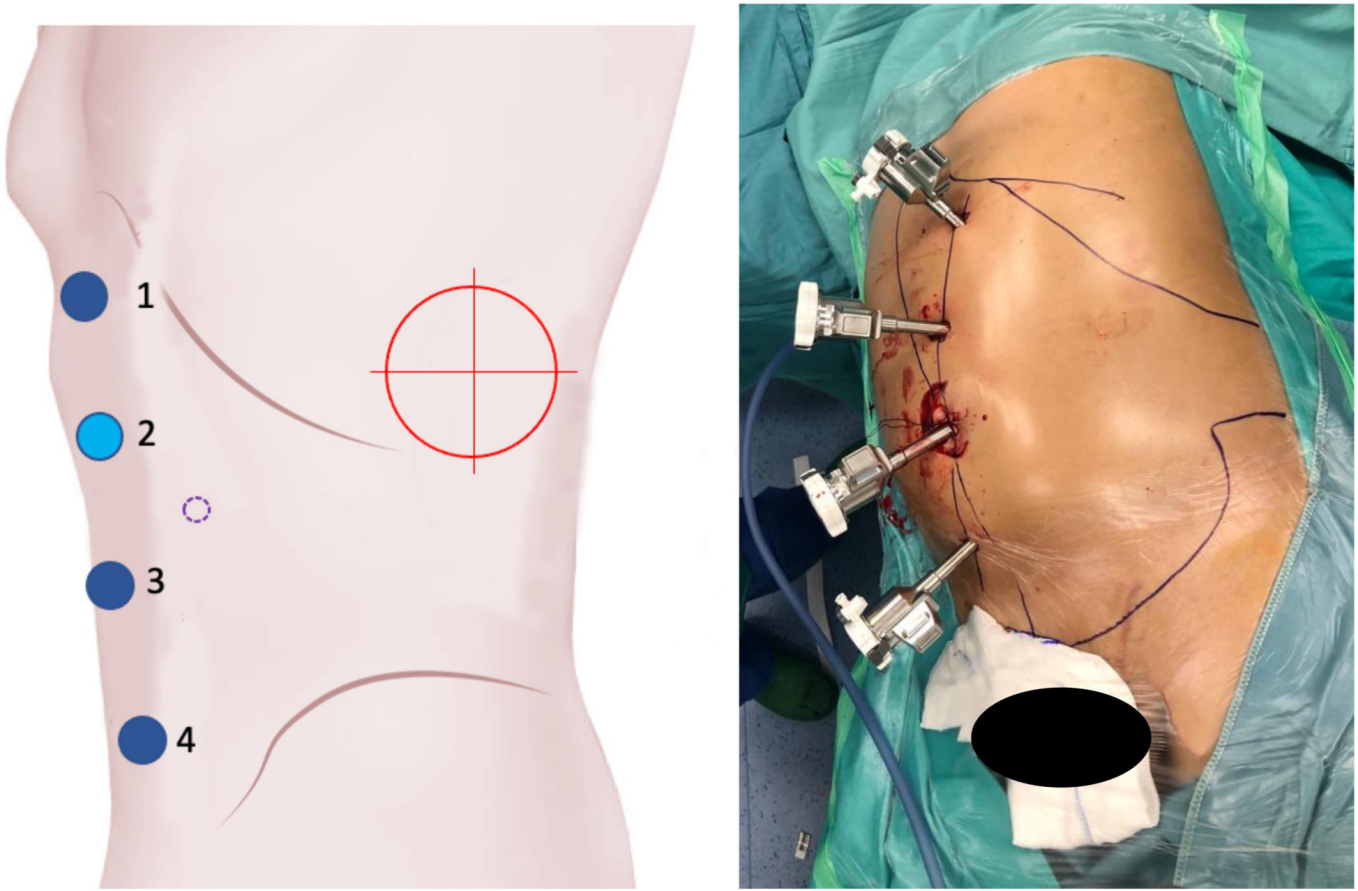
On the left, the port placement for surgeries involving the left kidney is shown. The light blue dot represents the camera, the dark blue dots indicate the operating instruments, the dot with a purple dotted line marks the assistant trocar, and the red target indicates the surgical objective. On the right, intraoperative photographs show the trocars in place prior to docking.

**Figure 5:**
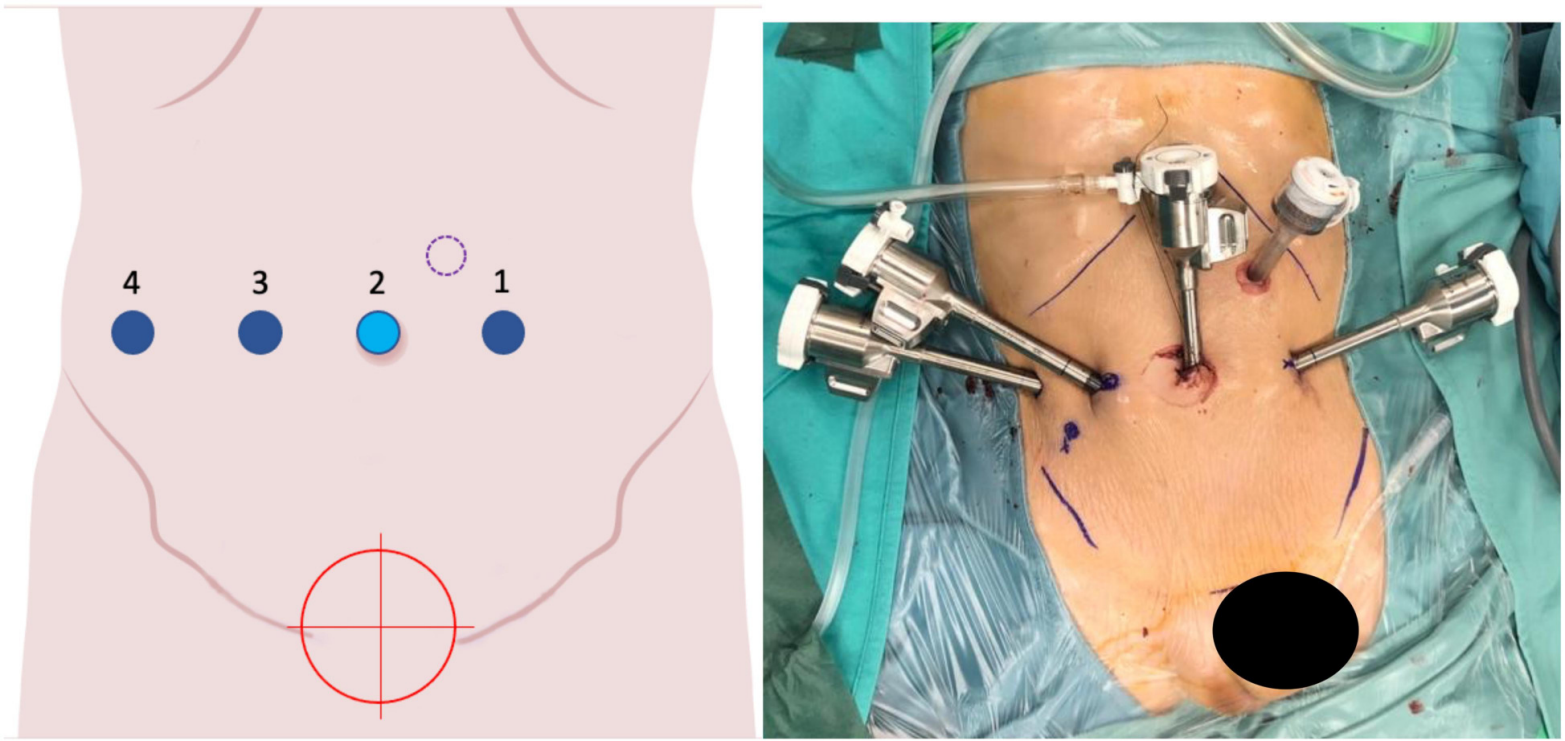
On the left, the port placement for pelvic surgeries (e.g., bladder, rectum) is shown. The light blue dot represents the camera, the dark blue dots indicate the operating instruments, the dot with a purple dotted line marks the assistant trocar, and the red target identifies the surgical objective. On the right, intraoperative photographs show the trocars in place prior to docking.

Prior to each robotic surgery procedure, the operating room layout was reviewed with the surgical team. Ensuring the optimal positioning of the robotic system components (console, patient, and video carts) is essential to provide sufficient space for all personnel (anesthetist, OR nurses, surgeons), enabling safe movement and easy access to the patient for routine or emergency maneuvers. Patient positioning and OR setup were standardized as much as possible and consistently applied across various robotic procedures. To facilitate this a two-dimensional 1:36 scale model of the operating room was created for each procedure, incorporating all components to determine the optimal configuration based on the scheduled procedure (Figures 1, 2). Furthermore, for each type of procedure, based on the anatomical site, diagrams have been created to indicate the optimal positioning of the trocars to perform the surgery (Figures 3–5).

### Statistical analysis

Descriptive statistics were expressed as percentages, with median and range or mean reported according to data variability. Chi-square tests were performed to evaluate the association between patient weight or ASA classification and the risk of conversion and postoperative complications.

## Results

### Patients' data and characteristics

A total of 569 pediatric patients underwent 569 procedures during the study period (328 males, 241 females). Of these, 509 were performed at IRCCS Giannina Gaslini (Genoa), and 60 at the other participating centers.

Median age at surgery was 73.8 months (range 4 months – 575.3 months). Median weight was 19.8 kg (range 5.8–133 kg). For 20 patients, the weight was not reported, these cases were therefore excluded from analyses involving it but were retained for all other applicable analyses.

Fifty-three (9.3%) patients weighed less than 10 kg (median weight: 8.5 kg). These patients underwent minimally invasive robotic surgery between 4 and 25 months of age. A total of 125 (21.9%) patients had a body weight between 10 and 15 kg, with ages ranging from 6 to 280 months.

In our series, 20.5% of patients had an ASA score greater than 2; specifically, 100 patients were classified as ASA III and 17 as ASA IV. For 21 patients, the ASA score was not reported; these cases were therefore excluded from analyses involving ASA classification but were retained for all other applicable analyses.

Console time data were missing for 55 patients; therefore, these cases were excluded from the analysis of console times but were retained for all other statistical analyses.

All patients' demographics and data are summarized in Table 1.

**Table 1:** Demographic data of the patients and operative time collected during 2015–2025.

Characteristics	Urology	Gastroenterology	Oncology	Thoracic and airways	Tot.
Patients	269	153	110	37	569
M:F	173:96	85:68	53:57	17:20	328:241
Median age at operation (range) [months]	47 (4.1–348)	124 (4–364)	93 (4–575.3)	100 (4–280)	73.8 (4–575.3)
Median weight (range) [Kg]	16 (6.1–80)	25 (7–133)	25 (5.8–96)	25.5 (7–76)	19.8 (5.8–133)
- Patients <10 kg	28 (6.1–9.6)	13 (7–9.5)	9 (5.8–9.6)	3 (7–9)	53 (5.8–9.6)
- Patients 10–15 kg	83 (10–14.5)	19 (10–14.5)	18 (10–14.5)	5 (10–11)	125 (10–14.5)
ASA score					
1	188	45	13	3	249
2	54	51	53	20	178
3	12	34	44	13	103
4	1	17	\	\	18
Duration of surgical procedure (range) [min]	135 (20–540)	190 (40–525)	175 (55–520)	160 (45–375)	160 (20–540)
Console time (range) [min]	80 (5–158)	90 (20–250)	90 (15–270)	107.5 (25–215)	87.5 (5–270)
Length of hospital stay [days]	3 (1–59)	7 (1–88)	5 (2–56)	6.6 (2–21)	5 (1–88)

All variables are expressed as median and range.

Series’ demographics and data according to different type of surgery.

ASA, American Society of Anaesthesiologists.

### Procedures

Each procedure subset was analyzed individually. Surgical time, console time, and hospital stay length by diagnosis and surgery type are summarized in Table 2 Intraoperative and postoperative complications are reported in Tables 3–6.

**Table 2:** List of robotic procedures.

Type of procedure	Numbers of procedures	Diagnosis
Urology Ureteral reimplantation: - dismembered (bilateral) - non dismembered (bilateral) Pyeloplasty: - dismembered - non dismembered Uretero-ureteral anastomosis Appendicovescicostomy/bladder augmentation Rectourethral fistula closure Heminephrectomy Nephrectomy Perirenal adhesiolysis Pyelotomy Urogenital reconstruction Gastrointestinal surgery Endorectal pull-through (Soave procedure) Ileocecal resection with ileocolic anastomosis Ileorectal anastomosis - with J-pouch - without J-pouch Fundoplication surgery (Nissen procedure) Esophagogastric dissociation Intestinal bypass Hepaticojejunostomy with Roux en-y technique Pancreatic cyst resection Abdominal cyst resection Multiple intestinal biopsies Rectal cuff division Cholecystectomy Rectal resection Gastric tansposition Heller myotomy Ladd's procedure Peritoneal adhesiolysis, gastropexy Colectomy Oncology Neuroblastic tumor resection - abdominal - thoracic Lymphadenectomy - abdominal - thoracic Sarcoma/teratoma resection - abdominal - thoracic Resection of metastasis - abdominal - thoracic Splenectomy Pancreatectomy (distal or DCP) Thymectomy Hysterectomy Resection of renal lesion Resection of liver neoplasm/biopsy Resection of thoracic cyst/biopsy Thoracic and airways Posterior tracheopexy Tracheal diverticulum resection Tracheal resection Mediastinal cyst Excision of bronchogenic cyst/extralobar sequestration Lobectomy Sympathectomy Esophagectomy, colon substitution Adhesiolysis of pleural adhesions Repair of diaphragmatic hernia	269 [110] 82(6) 28 (7) [110] 90 [15 redo] 20 15 7 1 6 10 6 3 1 153 19 14 [27] 22 5 34 14 8 4 1 13 1 2 5 1 1 5 2 1 1 110 57 41 16 5 4 1 3 2 1 4 2 2 17 6 6 1 5 4 2 37 17 2 1 2 3 3 1 1 1 6	53 VUR – 61 PM 10 UO - 3USt – 1UD 120 UPJO –1 HS – 5 BD– 1 ZS 6 RD – 2 ARM – 1 RCU – 5 EU 23 HSCR 23 UC – 15 CD 54 GERD/EIEE – 5 EA – 2 VACTERL 6 ChL – 16 AL 2 IM – 4 CC – 2 AMA - 1 SMAS 59 NB – 5 Lym 5 Sar – 16 Sph – 1 splenic hamartoma 6 PL – 9 AL – 2 TC – 6 Thy 1 UF 17 Tracheomalacia 2 TD – 1 TS – 9 TC – 1 EsSt 1 VT - 6 DH

VUR, Vescico-ureteral reflux; PM, primary obstructive megaureter; UO, Ureteral obstruction; 3USt , Ureteral stones; UD, Urethral duplication; UPJO, uretero-pelvic junction obstruction; HS, Hinman Syndrome; BD, bladder dysfunction; 1 Zinner Syndrome; RD, renal dysplasia; ARM, anorectal malformation; RCU, retro-caval ureter; EU, ectopic ureter; HSCR, Hirschsprung disease; UC, ulcerative colitis; CD, Crohn disease; GERD, gastroesophageal reflux; EIEE, early infantile epileptic encephalopathy; EA , esophageal achalasia; EA=esophageal atresia; ChL, cholelithiasis; IM, intestinal malrotation; CC, choledochal cyst; AL, abdominal lesion (Intestinal duplication, Lymphangioma, cystic lesion); AmA, aortomesenteric angle; SMAS, superior mesenteric artery syndrome; NB, Neuroblastoma; Lym, Lymphadenomegaly; Sar, sarcoma; Sph, Spherocytosis; PL, pancreatic lesion; TC, thoracic cyst; Thy, Thymoma; UF, Uterine fibromas; TD, Tracheal diverticulum; TS, Tracheal stenosis; EsSt, Esophageal stenosis; DH, diaphragmatic hernia; VT, Ventricular tachycardia.

**Table 3:** Urology - major intraoperative and post-operative complications.

Type of complications	Intraop.	Post-op.	CD	Treatment
Anastomotic leakage (urinary)	\	6	3b	2 Leakage drainage 3 Ureteral stent placement 1 Nephrostomy placement
Pelvic collection	\	3	3b	Surgical drainage
Incisional Hernia	\	3	3b	Manual hernia reduction
Ureteral obstruction	\	6	3b (5) – 4(1)	3 Ureteral stent placement 2 Nephrostomy placement 1 Ureterostomy
Intestinal perforation	\	1	3b	Primary repair of bowel perforation
Ureteral stent migration	\	1	3b	Ureteral stent placement
Displacement of the bladder catheter	\	1	3b	Placement of suprapubic catheter
Conversion to open for high risk of vascular injury	1	\	\	Conversion to open surgery
Conversion due to difficult ureteral dissection	1	\	\	Conversion to open surgery
Total	2 (0,7%)	21 (7.8%)		

**Table 4:** Gastrointestinal - major intraoperative and post-operative complications.

Type of complications	Intraop.	Post-op.	CD	Treatment
Minor bleeding	1	\	\	1 conversion to open
Duodenal obstruction	\	1	4	Reoperation (Duodenal- jejunal bypass redo)
Anastomotic leakage (GI tract)	\	6	3b	4 Ileostomy 2 Drainage of the collection
Pelvic collection	\	4	3b (3) – 4(1)	3 Surgical drainage 1 revision of the anastomosis and drainage of the collection assinstance
Conversion to open for high risk of vascular injury	1	\	\	Conversion to open
Evisceration of the omentum	\	1	3b	Reoperation
Conversion to open for intraoperative dissection difficulties	3	\	\	Conversion to open
Conversion to open for jejunal perforation	1	\	\	Conversion to open
Total	6 (3.9%)	12 (7.8%)		

**Table 5:** Oncology - major intraoperative and post-operative complications.

Type of complications	Intraop.	Post-op.	CD	Treatment
Minor bleeding	4	\	\	Conversion to open surgery
Bowel obstruction	\	1	4	Reoperation
Abdominal collection	\	5	3b (4) – 4 (1)	3 surgical drainage 1 ileostomy 1 Peritoneal toilette
Hemoperitoneum	\	1	3b	Peritoneal toilette
Central venous catheter infection	\	1	3b	Central venous catheter placement
Incisional hernia	\	2	3b	Manual hernia reduction
Bleeding and migration of a stone in the bile duct	\	1	3b	Embolization and endoscopic retrograde cholangiopancreatography
Spleen laceration	\	1	3b	Splenectomy
Conversion to open for high risk of vascular injury	4	\	\	Conversion to open surgery
Conversion due to difficulty in lesion isolation	19	\	\	Conversion to open surgery
Conversion caused by lesion infiltration	2	\	\	Conversion to open surgery
Conversion due to robotic malfunction	1	\	\	Conversion to open surgery
Total	30 (27,2%)	12 (11%)		

**Table 6:** Thoracic - major intraoperative and post-operative complications.

Type of complications	Intraop.	Post-op.	CD	Treatment
Oesophageal perforation	\	1	3b	Oesophageal stent positioning and chest drainage placement
Pneumothorax	\	1	3b	Chest drainage placement
Conversion to open for difficult dissection	4	\	\	Conversion to open surgery
Conversion due to intraoperative detection of right diaphragmatic hernia	1	\	\	Conversion to open surgery
Total	5 (13.5%)	2 (5,4%)		

### Urological procedures

A total of 269 (47.2%) urological procedures were performed. Two (0.7%) intraoperative complications occurred, both requiring conversion to open surgery. Twenty-one (7.8%) postoperative complications of grade ≥ 3b, according to the Clavien-Dindo classification, were reported.

### Gastrointestinal procedures

A total of 153 (26.9%) gastrointestinal (GI) procedures were performed. Twelve (7.8%) postoperative complications of grade ≥ 3b were recorded. Six (3.9%) conversions to either laparoscopy or open surgery were required.

### Oncological procedures

A total of 110 (19.3%) oncological procedures were performed during the study period. Thirty (27.2%) conversions to open surgery were necessary. Twelve (11%) postoperative complications of grade ≥ 3b were reported.

### Thoracic and airways procedures

A total of 37 (6.5%) procedures involving the thorax and airways were performed. Five (13.5%) conversions to either thoracoscopy or open surgery were required, and two postoperative complications (5.4%) of grade ≥ 3b occurred.

### Outcomes

Over the years, we have published several studies investigating the clinical outcomes of the surgical procedures, thereby enhancing the current evidence base regarding their effectiveness, safety profile, and long-term durability. When considered alongside findings from other independent research groups, these data offer a robust foundation for the evaluation of procedural performance. Some of these publications are summarized in Table 7.

**Table 7:** Selected publications by our group on robotic surgery (2015–2025).

Year	Authors	Title	Journal
2025	G. Mattioli et al.	From open to robotic surgery in pediatric ureteral reimplantation: overcoming the learning curve for improved outcomes	Frontiers in Surgery
2025	M.C.Y. Wong et al.	Laparoscopic robotic-assisted ileo-caecal resection with intracorporeal anastomosis in children with Crohn disease: initial experience of a paediatric center and surgical feasibility	Pediatric Surgery International
2024	M.C.Y. Wong et al.	Laparoscopic Robotic-Assisted Restorative Proctocolectomy and Ileal J-Pouch-Anorectal Anastomosis in Children: Shifting to a Two Stage-Approach	Journal of Laparoendoscopic & Advanced Surgical Techniques
2024	F. Palo et al.	Exploring the frontier in robotic pediatric cancer surgery: when to move forward and when to stop	Pediatric Surgery International
2023	V. Fiorenza et al.	Robotic approach to the distal ureter in Children: A survey from the European Association of Urology Robotic Urology Section Working Group for Reconstructive Urology	European Urology Open Science
2023	G. Mattioli et al.	Robotic ureteral reimplantation and uretero-ureterostomy treating the ureterovesical junction pathologies in children: Technical considerations and preliminary results	Journal of Robotic Surgery
2022	C. Romeo et al.	Laparoscopic robotic-assisted restorative proctocolectomy and ileal J-pouch-anorectal anastomosis in children	Pediatric Surgery International
2021	M. Torre et al.	Posterior tracheobrochopexy with thoracoscopic or robotic approach: technical details	Journal of Pediatric Endoscopic Surgery
2020	G. Mattioli et al.	Total oesophago-gastric dissociation in neurologically impaired children: Laparoscopic vs robotic approach	The International Journal of Medical Robotics and Computer Assisted Surgery
2017	G. Mattioli et al.	Robotic-Assisted Minimally Invasive Total Esophagogastric Dissociation for Children with Severe Neurodisability	Journal of Laparoendoscopic & Advanced Surgical Techniques
2017	G. Mattioli et al.	Da Vinci robotic surgery in a pediatric hospital	Journal of Laparoendoscopic & Advanced Surgical Techniques

This table presents a selection of peer-reviewed studies published by our group over the past decade, focusing on clinical outcomes, technical innovations, and comparative analyses in the field of robotic surgery.

## Discussion

The first pediatric case series on RS was published in 2001. Since then, RS has been applied to numerous gastrointestinal, genitourinary, and thoracic procedures in children (15–17). Most surgeries traditionally performed using conventional laparoscopic or thoracoscopic techniques have now been successfully replicated through a robotic approach. This trend has been largely driven by the growing number of urological procedures. Over time, an increasing number of complex reconstructive surgeries—such as Kasai portoenterostomy, Mitrofanoff Appendicovescicostomy, choledochal cyst excision, and tumor resections—have been performed successfully with robotic assistance, underscoring the continuous evolution and expanding capabilities of RS in pediatric care (15).

Comparative effectiveness studies on robotic pyeloplasty and gastric fundoplication have demonstrated that clinical outcomes are generally comparable to, or slightly superior to, those achieved with conventional techniques (15). However, for many low-volume procedures where robotic assistance is increasingly advocated, high-quality comparative effectiveness data may take considerable time to emerge.

A decade of experience at our center has confirmed the safety and efficacy of RS across a range of selected pediatric procedures. Despite its significant economic demands—especially in pediatric healthcare settings—we strongly advocate that centralization of care is essential to ensuring truly patient-centered management. In line with this principle, we have progressively and prospectively developed a dedicated robotic surgery program tailored to the pediatric population.

The robotic surgical system was initially introduced at our institution on a trial basis in 2015 for a period of 14 months. Subsequently, in March 2020, the system was permanently acquired for exclusive use within our pediatric hospital. During the interim period, two low-complexity cases were performed at an adult hospital, with patients transferred postoperatively via ambulance. This experience revealed significant logistical and clinical limitations, leading to the decision to fully integrate the robotic platform within our pediatric facility to ensure continuity of care and procedural sustainability.

Since March 2020, the permanent integration of the robotic platform at IRCCS Giannina Gaslini has enabled the safe and efficient execution of robotic procedures within the pediatric hospital itself. This has facilitated the management of more complex cases, eliminated the need for inter-hospital transfers, and ensured a higher standard of care in a dedicated pediatric setting.

Although this study shares some limitations with previous reports, it significantly expands the spectrum of surgical indications and case diversity, demonstrating safe and encouraging outcomes even in the youngest patients. We firmly believe that pediatric surgical care should be delivered by a dedicated multidisciplinary team within institutions specifically committed to pediatric healthcare—where quality, ethical standards, and the unique needs of children are prioritized.

1. Technology and safety

Compared to conventional laparoscopy, RS in adults offers several advantages, including enhanced ergonomics, tremor filtration, three-dimensional visualization, and more intuitive instrument control. However, early studies in the pediatric population raised concerns regarding the unique technical and safety challenges of this approach, such as limited intra-abdominal working space, which increases the risk of instrument collision or mechanical injury, restricted access to the abdominal cavity due to port positioning, and the relatively larger size of robotic ports and instruments in smaller patients (17).

Robot-assisted surgery is particularly well-suited for procedures requiring meticulous dissection and precise intracorporeal suturing. Optimal port placement must account for several critical factors: (a) patient size, (b) the location and dimensions of the target anatomical structure, and (c) the unique “geometry” of robotic systems, which favor a linear alignment of access points over the traditional triangulation used in conventional minimally invasive surgery. This conceptual shift allows for better planning of the number and positioning of robotic arms, minimizing both internal and external collisions during the procedure.

While it may seem intuitive that RS is ideally suited for the limited operative fields typical in pediatric patients, current RS systems and instruments are not specifically tailored for pediatric anatomy. Technical constraints may render their application challenging—or even unfeasible—in smaller patients. For example, the manufacturer of the da Vinci® surgical system recommends a minimum inter-port distance of 8 cm, which is often difficult to achieve in neonatal and infant cases. Instrument size and length also represent significant limitations. Neonatal procedures are typically performed using 3-mm instruments and endoscopes, which are substantially smaller than those currently available for robotic platforms. The absence of commercially available 3-mm robotic instruments remains a major constraint, limiting the applicability of robotic systems in neonates and small infants and hindering their widespread use in this population (18).

Furthermore, the da Vinci® system features integrated fluorescence imaging through near-infrared (NIR) technology—Firefly®—which enhances intraoperative visualization of vascular and anatomical structures. This technology utilizes fluorescent compounds—most commonly indocyanine green (ICG)—which, when activated by a near-infrared (NIR) light source at a wavelength of approximately 820 nm, emit fluorescence detectable only through dedicated imaging systems. The resulting images are particularly valuable in hepatobiliary surgery for the identification of bile ducts and vascular structures. Moreover, the application of this modality is expanding in pediatric oncologic surgery, where it shows promise in delineating tumor margins, identifying vascular anatomy, detecting metastases, guiding sentinel lymph node biopsies, preserving adjacent critical structures, and assisting in reconstructive steps. In pediatric urology, ICG fluorescence is primarily used intraoperatively to assess renal perfusion, particularly during pyeloplasty in cases of hydronephrosis secondary to crossing vessels (13, 14, 19, 20).

2. Patient weight, surgical complexity and clinical considerations

**Figure 6:**
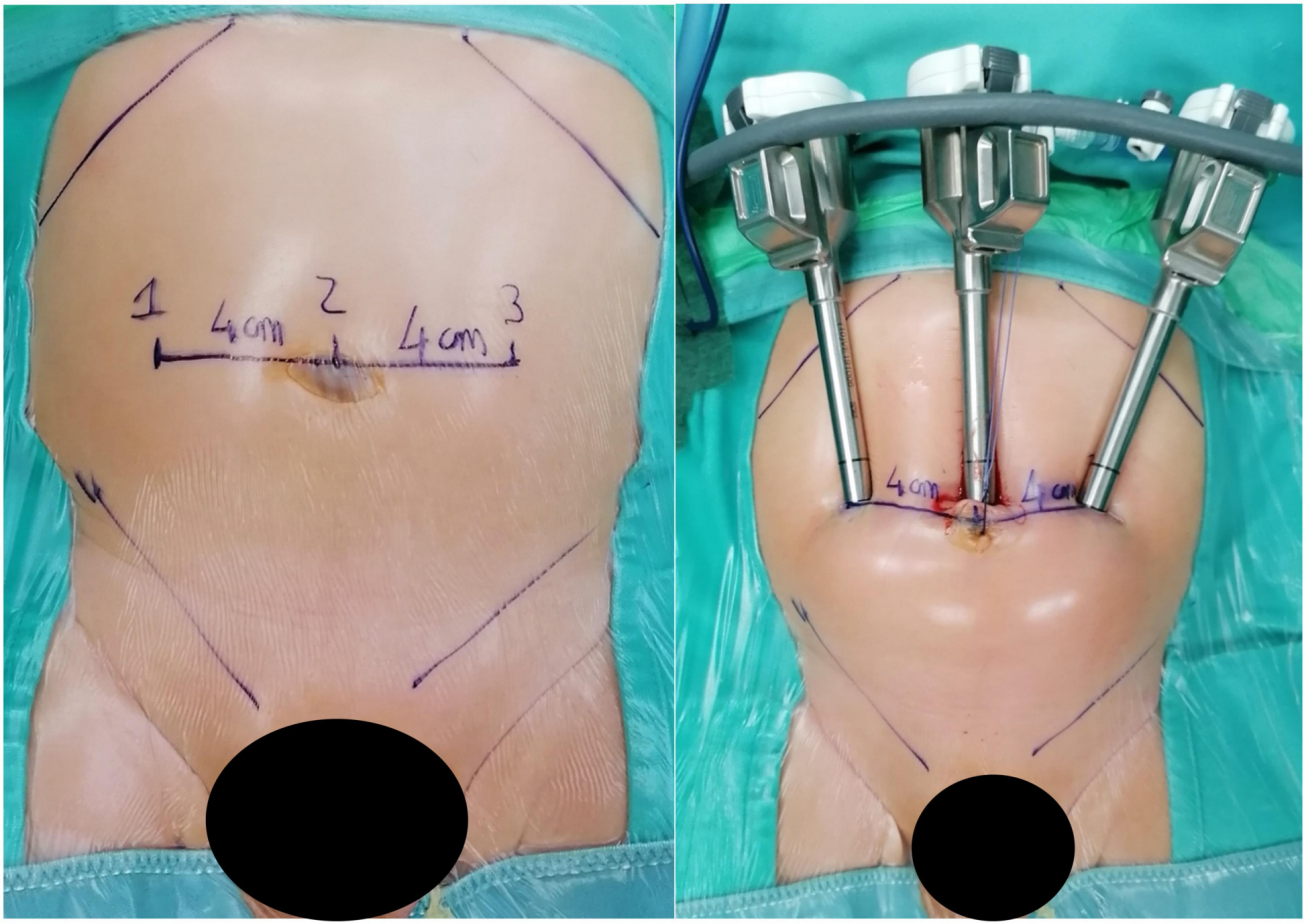
On the left, the measurement of the distance between trocars in a 7 kg patient with bilateral vesicoureteral reflux is shown. On the right, the image taken after trocar placement is displayed. The average distance between the trocars is approximately 4 cm.

**Figure 7:**
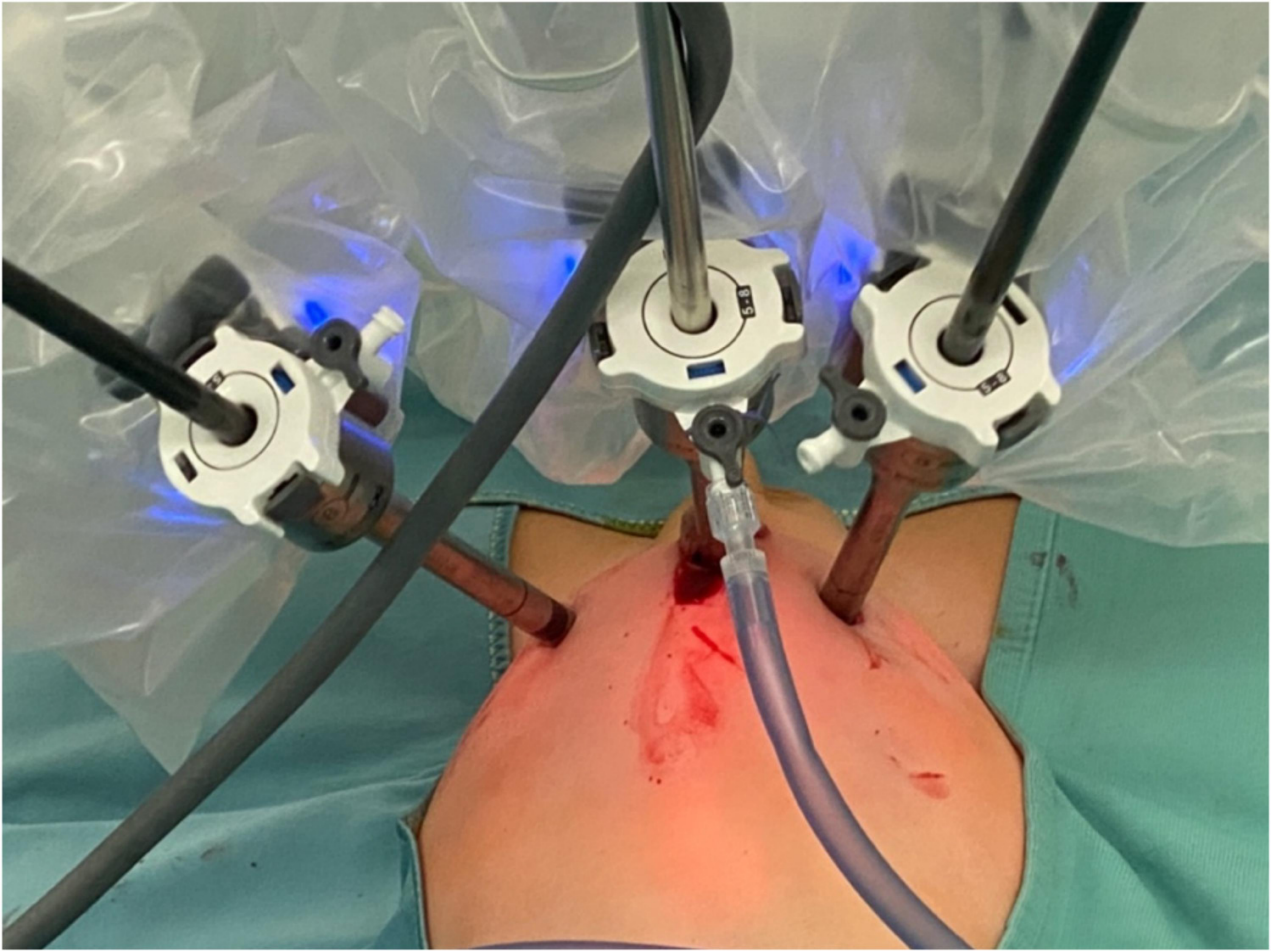
Intraoperative photo showing absence of external conflicts between trocars of the robotic arms.

In our cohort, 117 patients (20.5%) had an ASA score of ≥3, indicating a significant level of clinical complexity. These patients often presented with chronic cardiac or pulmonary conditions that require enhanced perioperative management, especially from an anesthesiological standpoint. Moreover, the size and positioning of the robotic system—particularly in pediatric patients—significantly restrict access to the airway, creating additional challenges for anesthesiologists to perform essential maneuvers for maintaining adequate respiratory support. The use of the robotic platform also necessitates prolonged operative times and specific patient positioning, which further increase anesthetic complexity. Limited physical access due to the robotic arms can delay or complicate urgent airway interventions, making meticulous preoperative planning and intraoperative vigilance essential. Altogether, these factors contribute to the increased perioperative risk observed in patients with higher ASA scores undergoing robotic-assisted surgery, especially in the pediatric population. These findings support the feasibility and safety of robot-assisted surgery, even in high-risk pediatric populations. Based on our experience, maintaining a 3–4 cm distance between trocars has proven feasible in both thoracoscopic and laparoscopic procedures, without resulting in conflicts between robotic arms (Figures 6, 7).

Preoperative planning is essential to ensure the selection of instruments tailored to each procedure, thereby minimizing material waste and reducing the risk of conversion to open surgery. Even in smaller patients, we opted for the use of four trocars, maintaining inter-trocar distances of less than 4 m when necessary. This configuration allowed us to fully exploit the advantages of an additional robotic arm, thereby avoiding the need for an accessory trocar, which can be ergonomically challenging for the bedside assistant in certain scenarios. This approach also enhances control for the console surgeon, improving operative precision and efficiency.

In our cohort, 53 patients (9.3%) weighed less than 10 kg, with ages ranging from 4 to 25 months. Patients weighing between 10 and 15 kg accounted for 21.9% of the cohort. These findings suggest that low body weight (except when less than 5 g) is not a contraindication for robotic surgery.

We performed an analysis to assess whether there was an association between patient weight categories and the likelihood of conversion from robotic to open surgery. Patients were categorized into three weight groups (<15 kg, 15–30 kg, >30 kg). The Chi-square test showed no statistically significant association between these weight categories and conversion rates (*χ*^2^ = 1.60, *p* = 0.45), indicating that conversion rates do not differ significantly across the defined weight groups.

3. Complications and Conversions

The overall conversion-to-open procedure rate across urologic, gastrointestinal, and thoracic surgeries was 2.12%. These findings are consistent with those reported in a meta-analysis of pediatric robotic surgery, which indicated conversion rates of 3.9%, 1.3%, and 10% for gastrointestinal, genitourinary, and thoracic procedures, respectively (18). Specifically, urologic procedures had a conversion rate of 0.7%, gastrointestinal procedures had a rate of 3.9%, and thoracic procedures had a rate of 10.8%.

The Chi-square test revealed a statistically significant association between patient complexity category and the likelihood of conversion from robotic to open surgery (*χ*^2^ = 33.61, *p* < 0.001), indicating that the conversion rate varies significantly across complexity levels.

Our patient series confirms that gastrointestinal, thoracic, and urological reconstructive procedures can be successfully completed using robotic surgery. However, complex oncologic procedures, particularly those involving Image-Defined Risk Factors (IDRFs), are more likely to require conversion, with a conversion rate approaching 27.7% in our series.

Oncological procedures that required conversion to open surgery were, in most cases, not due to emergency situations, but rather due to significant anatomical alterations—especially in patients who had previously undergone radiotherapy, chemotherapy, or prior surgery. The decision to convert to open surgery was influenced by several factors, most commonly the complexity of vascular management. Additionally, conversions were sometimes required due to indistinct tumor margins or the presence of adhesions complicating the procedure. Based on this experience, it can be suggested that tumors subjected to preoperative treatments present challenges for minimally invasive surgery.

Postoperative major complications occurred in 47 out of 569 cases, representing a rate of 8%. The early complication rate observed in our patient series is consistent with previously published data, which report an incidence ranging between 2.5% and 8% (10, 15).

The Chi-square test did not reveal a statistically significant association between ASA class and the occurrence of postoperative complications (*χ*^2^ = 5.69, *p* = 0.13).

4. Laparoscopic vs. robotic surgery

The advent of robotic surgical systems has significantly impacted minimally invasive reconstructive procedures. Many operations traditionally performed via laparoscopy have been increasingly replaced by robotic techniques, which have expanded from urological and gastrointestinal surgeries to thoracic and oncological interventions.

A multicenter study involving 322 patients undergoing pyeloplasty reported that operative time was significantly shorter in open procedures compared to laparoscopic and robot-assisted laparoscopic approaches (*p* < 0.0001). The duration of hospital stay was reduced in the robotic-assisted group relative to the other techniques (*p* < 0.0001) (21).

Additionally, robotic-assisted nephrectomy has proven to be a valid and efficient alternative to conventional laparoscopic nephrectomy in managing renal duplication. Although robotic-assisted surgery generally incurs higher hospitalization costs, primarily attributable to equipment and technology, it demonstrates comparable overall cost-effectiveness with traditional laparoscopy (22). The literature indicates that robotic-assisted nephrectomy is associated with fewer complications, decreased postoperative pain, and shorter recovery periods, potentially reducing healthcare expenses and improving patient outcomes.

Other studies comparing gastrointestinal surgical approaches have evaluated standard laparoscopy against robotic surgery. A meta-analysis on the surgical treatment of Hirschsprung's disease (HSCR) confirms that RS appears to be a safe alternative to laparoscopy in pediatric patients. Four studies, totaling 291 pediatric patients (124 treated with robotic-assisted surgery and 167 with laparoscopic-assisted surgery), demonstrated that RS was associated with significantly reduced intraoperative blood loss (23). However, this benefit was counterbalanced by notably higher hospitalization costs.

Initial outcomes of the robotic-assisted Heller-Dor procedure for pediatric achalasia suggest several advantages over conventional laparoscopy, including reduced blood loss (23 ± 15 ml vs. 95 ± 15 ml; *p* < 0.001) and shorter operative time (178 ± 25 min vs. 239 ± 55 min; *p* = 0.009) (24). Notably, the RS group exhibited no recurrences, compared to five in the laparoscopic group (*p* = 0.039), a significantly lower reintervention rate (0% vs. 41.7%; *p* < 0.039), and markedly reduced overall healthcare costs ($28,660 vs. $60,360; *p* < 0.001) (24).

The clinical benefits of robotic surgery over laparoscopy become more evident in complex procedures. A study involving 90 pediatric patients (66 females and 24 males) comparing robotic (*n* = 20) and laparoscopic (*n* = 70) approaches for Roux-en-Y hepaticojejunostomy in cases of choledochal cysts found that RS yielded significantly lower intraoperative blood loss and shorter hepaticojejunostomy completion times (58 ± 12 min vs. 71 ± 11 min; *p* < 0.001) (25). However, the total operative time was longer in the robotic group (284 ± 14 min vs. 195 ± 18 min; *p* < 0.001). The robotic approach provided improved ergonomics and superior surgical conditions, supporting the integrity of the hepaticojejunostomy, which is essential for surgical success.

5. Training, research and economic aspects

Important questions remain regarding the learning curve and skill acquisition for surgeons. As with many innovative technologies, the adoption of robotic surgery (RS) has not been universal. Similar to conventional minimally invasive surgery, robotic-assisted techniques initially faced skepticism and challenges, requiring time to demonstrate their safety, reproducibility, and clinical value.

Some authors suggest that robotic surgery has achieved wider adoption more rapidly than traditional laparoscopy, primarily due to its ability to facilitate complex procedures with a shorter learning curve (26, 27). Enhanced wrist articulation, high-definition magnification, and three-dimensional visualization have transformed the laparoscopic landscape. These technological advances significantly reduce the number of cases required to achieve surgical proficiency, allowing even less experienced laparoscopists to perform advanced minimally invasive procedures with high precision and consistency (28).

However, this rapid adoption raises concerns, particularly regarding the potential for increased complication rates as access to robotic systems becomes more widespread. This is especially relevant in highly complex neonatal procedures, such as tracheoesophageal fistula repair, where the rarity of the condition limits the number of cases a surgeon can perform annually, making it difficult to overcome the steep learning curve.

The introduction of new surgical technologies not only brings novel tools but also introduces new paradigms in knowledge acquisition, cognitive processing, and intraoperative decision-making. It demands enhanced situational awareness, evolving team dynamics, and continuous reassessment of operative strategies (28). While robotic systems offer substantial advantages, their integration into clinical practice carries inherent risks—such as inefficiency, increased procedural complexity, and potential harm—if not carefully managed.

Economic considerations, primarily evaluated through incurred costs and revenues generated from national health system reimbursements, currently represent one of the main barriers to the widespread adoption of robotic surgery. Moreover, traditional cost-benefit analyses based solely on financial statements are often inadequate for evaluating innovative technologies with significant social and ethical implications. This is due to the limitations of purely accounting-based methods in capturing broader social impacts on healthcare organizations and their stakeholders.

As part of the Health Technology Assessment (HTA) throughout the entire life cycle of the technology and procedure, and with the goal of improving robotic surgery outcomes—including from an economic perspective—it may be both important and beneficial to enhance operating room planning in terms of staffing, scheduling, and procedural estimation. These improvements could help optimize resource utilization and support better negotiation of material costs.

The clinical benefits highlighted in this paper (and in the references) support the use of robotic systems in pediatric surgery and open the door for more comprehensive cost-benefit evaluations of this innovative technology.

In particular, it will be valuable to explore both HTA dimensions—such as safety, clinical effectiveness, economic evaluations, and organizational, ethical, legal, and social aspects—and the Social Return on Investment (SROI), a framework designed to measure the total social value created by an activity in relation to the costs incurred. Such analyses will allow for a more holistic understanding of the impact of robotic surgery and enable the development of robust proposals to revise diagnosis-related group (DRG) reimbursement rates for pediatric robotic interventions. This, in turn, would support the assessment of both financial and social returns, ultimately guiding more ethical and sustainable healthcare policies.

## Conclusion

Pediatric RS marks a significant advancement in the evolution of minimally invasive surgical techniques. Our data suggest that RS provides substantial advantages in terms of precision, visibility, and control during complex procedures, even in pediatric patients with lower age and weight. The centralization of resources in specialized centers is crucial for optimizing outcomes and ensuring the safety and effectiveness of pediatric robotic surgery.

## Data Availability

The raw data supporting the conclusions of this article will be made available by the authors, without undue reservation.
